# CD200/CD200R: Bidirectional Role in Cancer Progression and Immunotherapy

**DOI:** 10.3390/biomedicines11123326

**Published:** 2023-12-16

**Authors:** Christopher Nip, Leyi Wang, Chengfei Liu

**Affiliations:** 1 Department of Urologic Surgery, University of California, Davis, CA 95817, USA; ccnip@ucdavis.edu (C.N.); lcdwang@ucdavis.edu (L.W.); 2 Graduate Group in Integrative Pathobiology, University of California, Davis, CA 95817, USA; 3 UC Davis Comprehensive Cancer Center, University of California, Davis, CA 95817, USA

**Keywords:** cancer, CD200, CD200R, immunosuppressive signaling, tumor immune microenvironment, checkpoint, therapy

## Abstract

As an immune checkpoint molecule, CD200 serves a foundational role in regulating immune homeostasis and promoting self-tolerance. While CD200 expression occurs in various immune cell subsets and normal tissues, its aberrant expression patterns in hematologic malignancies and solid tumors have been linked to immune evasion and cancer progression under pathological conditions, particularly through interactions with its cognate receptor, CD200R. Through this CD200/CD200R signaling pathway, CD200 exerts its immunosuppressive effects by inhibiting natural killer (NK) cell activation, cytotoxic T cell functions, and M1-polarized macrophage activity, while also facilitating expansion of myeloid-derived suppressor cells (MDSCs) and Tregs. Moreover, CD200/CD200R expression has been linked to epithelial-to-mesenchymal transition and distant metastasis, further illustrating its role in cancer progression. Conversely, CD200 has also been shown to exert anti-tumor effects in certain cancer types, such as breast carcinoma and melanoma, indicating that CD200 may exert bidirectional effects on cancer progression depending on the specific tumor microenvironment (TME). Regardless, modulating the CD200/CD200R axis has garnered clinical interest as a potential immunotherapeutic strategy for cancer therapy, as demonstrated by early-phase clinical trials. However, further research is necessary to fully understand the complex interactions of CD200 in the tumor microenvironment and to optimize its therapeutic potential in cancer immunotherapy.

## 1. Introduction

Immune checkpoint molecules maintain immune homeostasis by orchestrating a balance between stimulatory and inhibitory signals that are responsible for mounting immune responses and preserving self-tolerance. These immune checkpoint molecules, such as PD-1, LAG-3, CTLA-4, and CD200, are commonly expressed on activated T cells and have been reported to modulate T cell activity and prevent autoimmunity [1,2]. There are various innate and adaptive immune cells in tumors and their environments whose signaling and metabolism significantly affect tumor progression. Previously, immune checkpoint tumoral expression has been proven to suppress the immune response and facilitate cancer progression [3].

The blockade of these immune checkpoint molecules has been theorized to abrogate T cell immunosuppression and reactivate T cell responses, enhancing antitumor immunity. This novel approach towards cancer therapy has been well studied using anti-PD-1/PD-L1 and anti-CTLA-4 inhibitors with durable response rates in patients with various cancer types, and research has extended towards blockade of other tumor-associated immune checkpoint molecules, such as CD200, involved in modulating the immune response, including the combination of immune checkpoint blockade therapies with other treatments [4,5,6,7].

CD200 is upregulated in various cancer types and associated with immunosuppression. However, there is evidence suggesting that CD200 may also contain anti-tumor effects based on its suppression of tumor-promoting inflammation, angiogenesis, expansion of tumor-associated myeloid cells (TAMC), and improvement of the efficacy of anti-PD-1/PD-L1 treatments [8,9,10,11,12,13,14,15,16,17]. This literature review will provide an overview of the mechanism of the CD200/CD200R axis and highlight significant findings of its bidirectional role in carcinogenesis, along with its clinical relevance in immunotherapy and targeted therapy.

## 2. Biology of the CD200/CD200R Axis, Expression Patterns, and Signaling in Cancer

CD200, alternatively known as OX-2, is a single-pass, type I membrane glycoprotein that belongs to the immunoglobulin superfamily (IgSF) of proteins. It contains two extracellular immunoglobulin-like domains, a transmembrane domain, and a cytoplasmic 19 amino-acid “tail” [18]. CD200 expression has been detected in a wide array of immune cells and normal tissues, including human thymocytes, neurons, activated T cells, B cells, dendritic cells, vascular endothelial cells, kidney glomeruli, epithelial keratinocytes, and syncytiotrophoblasts. Recent studies have also reported CD200 expression across hematologic malignancies, solid tumors, and skin cancers. CD200 binds to its cognate receptor, CD200R, which is highly expressed in the tumor microenvironment (TME) on TAMCs, including tumor-associated macrophages (TAMs), myeloid-derived suppressor cells (MDSCs), and tumor-associated dendritic cells (TADCs) [8]. CD200R expression also occurs in monocytes, macrophages, neutrophils, dendritic cells, mast cells, B lymphocytes, and a subset of T lymphocytes. CD200R is also an IgSF protein and contains two extracellular Ig-like domains. However, its cytoplasmic 67 amino acid “tail” possesses three phosphorylatable tyrosine residues that participate in intracellular signaling.

**Figure 1:**
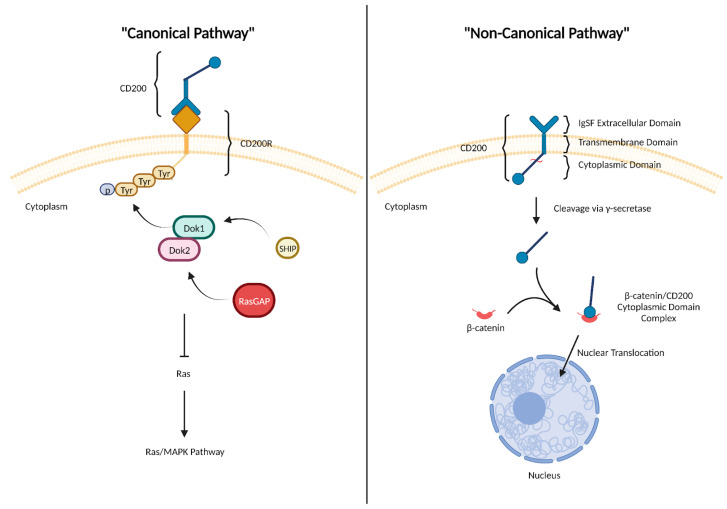
**CD200/CD200R signaling: “Canonical” and “Non-Canonical” pathways**. Through its “Canonical” pathway, CD200 exerts its downstream immunosuppressive effects after interacting with its cognate receptor, CD200R. This causes the recruitment of adaptor proteins—Dok1, Dok2, SHIP, and RasGAP—and inhibits Ras/MAPK signaling. CD200 also participates in a “non-canonical” intracellular pathway that promotes a pro-tumor phenotype, independently of signaling through CD200R. “https://www.biorender.com/ (accessed on 27 November 2023)”.

CD200 exerts its effects primarily through engagement with CD200R, often referred to as its “canonical” action as an immune checkpoint molecule (Figure 1). Upon CD200/CD200R interaction, the third tyrosine residue, located within the NPXY motif, becomes phosphorylated and recruits the adaptor proteins, Dok-1 and Dok-2. These proteins subsequently become phosphorylated and bind SH2-domain-containing proteins, including Ras GTPase activating protein (RasGAP) [19]. This Dok-2/RasGAP complex inhibits the activation of Ras. Dok-1 also interacts with and recruits tyrosine-phosphorylated SHIP—though Dok-1/SHIP activity has no significance in mediating downstream CD200R effects, as demonstrated through knockdown experiments by Mihrshahi et al. [20]. Collectively, these effects culminate in the downregulation of the Ras/MAPK pathway and reduced activation of ERK, JNK, and p38 MAPKs in mast cells [19]. This downstream signaling activity impairs cytokine production and mast cell degranulation, illustrating how CD200/CD200R signaling exerts an inhibitory effect on myeloid cell function.

Recently, a “non-canonical” role for CD200 has also been reported where it triggers an intracellular signaling pathway that functions independently of CD200R (Figure 1). In this pathway, γ-secretase induces cleavage of the CD200 cytoplasmic domain, which was previously believed to be signaling-inert. This cleaved CD200 cytoplasmic domain can subsequently bind β-catenin and translocate into the nucleus where it directly enhances the expression of transcription factors known to be associated with cell proliferation and cancer progression [21,22,23].

## 3. Bidirectional Role of CD200/CD200R Signaling

The CD200/CD200R axis serves a pivotal role in maintaining immune tolerance and protecting healthy tissues from needless immune damage. It is believed to modulate levels of activated myeloid cells in normal and pathological conditions, preventing chronic inflammation and autoimmune disease. It also serves a central role in balancing adequate immunosurveillance, inflammatory responses, and pathogen removal, while also preserving tissue homeostasis. However, CD200 expression has also been implicated in the pathogenesis of solid tumors and hematologic malignancies by mediating immunosuppression of the innate and adaptive immune responses. CD200 expression has been well documented to exert adverse effects on clinical outcomes and has been identified as a negative prognostic factor for patients—a finding that persists across various lymphoproliferative disorders, solid tumors, and skin cancers. For instance, in a cross-sectional study of 67 patients with mature B cell lymphoproliferative disorders, high levels of CD200 expression were correlated with advanced stage on the Rai and Binet staging systems along with earlier time to progression in CLL (Chronic Lymphocytic Leukemia) patients [24]. In pediatric B cell acute lymphoblastic leukemia (B-ALL), Aref et al. reported decreased responses to remission-induction therapy for CD200+ cases (19/28 or 67.8%) relative to CD200− cases (14/15 or 93.3%) along with significantly shorter overall survival (OS) and disease-free survival (DFS) [25]. For patients with cytogenetically normal acute myeloid leukemia(AML), high CD200 expression was associated with lower complete remission rates (9/18 or 50%) relative to CD200− patients (56/71 or 79%) and was accompanied by lower 3-year DFS (0%) and 3-year overall survival (0%) relative to CD200− patients (65% and 51%, respectively) [26]. For patients with colorectal liver metastasis, high CD200 expression was associated with significantly poorer prognosis (5-year survival rates of 26.9%) relative to low CD200 expression (5-year survival rates of 54.1%) [27]. In bladder cancer, Rexin et al. reported an association between CD200 expression and tumor grade [28]. Similarly, in cutaneous squamous cell carcinoma, CD200 expression levels were associated with tumor grade and clinical stage where patients with high CD200 expression had shorter overall survival (31.3 months) relative to patients with low expression (41.9 months) [29]. These studies collectively suggest that CD200 expression serves a pivotal role in facilitating cancer progression.

### 3.1. CD200/CD200R Signaling Promotes Tumor Progression

#### 3.1.1. Suppression of Natural Killer Cells

**Figure 2:**
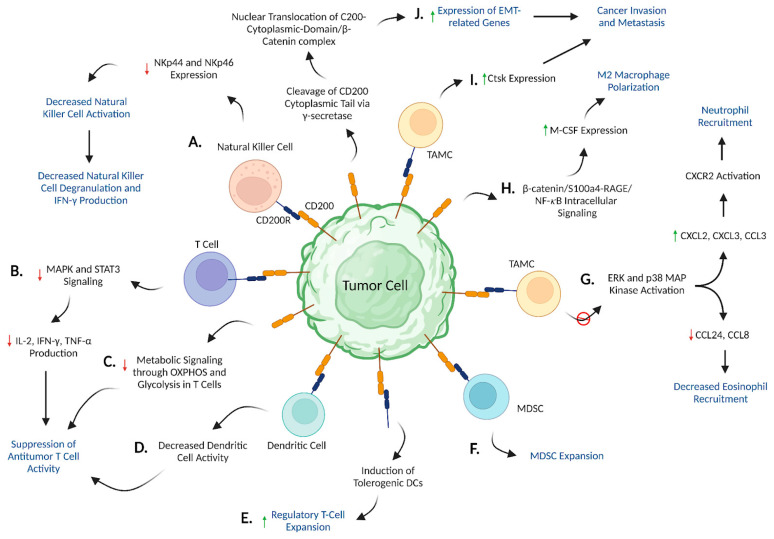
**Proposed mechanisms elucidating the pro-tumorigenic role of CD200/CD200R signaling in cancer progression**. CD200/CD200R signaling promotes an immunosuppressive TME by (**A**) inhibiting NK cell activation through decreased NKp44 and NKp46 expression, (**B**) reducing MAPK/STAT3-mediated Th1 cytokine production, (**C**) hampering metabolic signaling within T cells leading to an inactive T cell phenotype, (**D**) suppressing DC-mediated T cell activation, facilitating expansion of (**E**) Tregs and (**F**) MDSCs, (**G**) differentially regulating production of chemokines involved in immune cell recruitment, for example, CXCL2, CXCL3, CCL3, CCL24, and CCL8 by inhibiting ERK and p38 MAP kinases, and (**H**) driving M-CSF-mediated M2 macrophage polarization through increased β-catenin/S100a4-RAGE/NF-𝜅B/M-CSF signaling. Furthermore, CD200/CD200R signaling stimulates cancer invasion and metastasis by increasing the expression of (**I**) Ctsk and (**J**) EMT-related genes. “https://www.biorender.com/ (accessed on 22 September 2023)”.

CD200 expression on human cancer cells is believed to confer a pro-tumorigenic role in cancer development by fostering the formation of an immunosuppressive tumor microenvironment. One such proposed property of CD200 is the suppression of natural killer (NK) cell anti-tumor responses via engagement with CD200R on NK cells. NK cells are effector immune cells of the innate immune system that release cytotoxic products, including perforins and granzymes, to kill malignant and virus-infected cells, particularly during the early immune response [30,31,32]. These immune cells are activated by a collection of cytokines and natural cytotoxicity receptors (NCRs), including NKp30, NKp44, and NKp46 which deliver stimulatory signals after recognizing malignant cell markers [33,34,35]. Interestingly, CD200 is believed to exert its immunosuppressive effects on NK cells by negatively regulating these NCR expression levels (Figure 2A). In a study involving patients with AML, high CD200 expression was associated with significantly decreased levels of NKp44 and NKp46 in NK cells. These reduced NCR levels interfere with NK cells’ cytolytic activity, which was reflected by their diminished capacity for degranulation. Furthermore, NK cells serve an immuno-modulatory role as a primary producer of IFN-γ, which functions to activate T cells [36,37]. However, patients with high CD200 expression showed a significant reduction in NK cells producing this cytokine, which further suppressed cell-mediated immunity. The administration of anti-CD200 to block CD200/CD200R signaling reversed these effects by increasing the frequency of NK cell degranulation and augmenting IFN-γ production. Taken together, these findings suggest a direct role of CD200 in inhibiting NK cell activity through interactions with CD200R.

#### 3.1.2. Regulation of T Cell Function

Similarly, the CD200/CD200R axis has also been documented to suppress the adaptive immune response by inhibiting effector and memory T cell anti-tumor activity. As demonstrated in AML studies [38], CD200 expression has been associated with reduced levels of CD4+ and CD8+ memory T cells along with a concomitant decrease in the production of pro-inflammatory Th1 cytokines, including TNF-α, IL-2, and IFN-γ. This pattern of dampened T cell cytokine release is a byproduct of inhibited MAPK and STAT3 signaling pathways (Figure 2B). Specifically, following engagement between CD200 and CD200R, the Dok2 adaptor protein recruits and interacts with the SH2 domain on RasGAP. This Dok2/RasGAP complex subsequently inhibits Ras activation and disrupts signaling through the Ras/MAPK pathway, negatively regulating cytokine production [19,20]. STAT3 signaling also modulates cytokine release and was similarly found to be suppressed by CD200/CD200R interactions [39].

Furthermore, CD200 expression has been associated with impaired T cell activity and metabolic functions [20]. Using in vivo AML models, CD8+ memory T cells isolated from CD200+ tumors appeared to be immunophenotypically inactive as characterized by the absence of CD69 and CD127—markers indicative of T cell activation. These immune cell subsets also lacked Ki-67 expression, a marker of cell proliferation. Both of these processes—T cell activation and proliferation—are inherently dependent on increases in oxidative phosphorylation and glycolysis to supplement the heightened energy demands [40]. However, T cells isolated from CD200+ AML tumors possessed significantly downregulated genes involved in metabolic signaling compared to CD200− tumors (Figure 2C). This indicates a potential CD200-mediated suppression of T cell metabolism, thus indirectly compromising these immune cells’ cytotoxic functions, ability to maintain tumor immunosurveillance, and maintenance of prolonged anti-tumor/anti-viral responses [41]. Further research is necessary to determine whether this inhibition operates through CD200R signaling or other CD200-dependent secondary mechanisms.

CD200/CD200R signaling also exerts indirect immunosuppressive effects on effector T cell activity by repressing dendritic cell (DCs) function. DCs serve as antigen-presenting cells (APCs) that prime naïve CD4+ and CD8+ T cells using major histocompatibility (MHC) molecules and therefore heavily influence the magnitude and quality of T cell responses [42]. Petermann et al. demonstrated that CD200 expression in melanoma cell lines was associated with an inability of DCs to activate primary T cells (Figure 2D), as evidenced by significantly decreased IL-2 and IFN-γ production in mixed lymphocyte reactions (MLR). shRNA knockdown of CD200 reversed these immunosuppressive effects and restored Th1 cytokine production, further supporting the role of CD200 in impairing T cell activity via DC inhibition [43].

#### 3.1.3. Expansion of Regulatory T Cells

CD200/CD200R signaling has been posited to facilitate cancer progression through the recruitment and tumor infiltration of regulatory T cells (Tregs). Regulatory T cells function to modulate the immune response and maintain self-tolerance through CTLA-4-mediated suppression of T cell activity, the production of immunosuppressive cytokines, including IL-10, IL-35, and TGFβ, and the release of granzymes and/or perforins to target effector cells [44,45,46,47,48,49,50]. These functions collectively serve to inhibit anti-tumor immunity, thereby preventing adequate immune surveillance of cancer and hampering effective anti-tumor immune responses. Thus, it is no surprise that Tregs have been associated with poor prognoses in AML [51,52]. Moreover, as demonstrated by several correlational studies, the frequency and tumor infiltration of Tregs significantly positively correlates with CD200 expression, which indicates a pathophysiologic link between CD200 activity and Treg induction [53,54,55]. Clinical and in vitro studies reinforce this assertion as the administration of an anti-CD200 monoclonal antibody to block CD200/CD200R signaling was shown to be sufficient in reducing Treg frequencies for chronic lymphocytic leukemia patients and cell samples [55,56]. Studies speculate that the mechanism underlying this correlation involves the differentiation of bone marrow stem cells towards suppressive and tolerogenic DCs, which preferentially cause induction of Tregs (Figure 2E) [55,57,58,59]. This process is believed to be mediated by the CD200/CD200R interaction. Furthermore, in AML studies, these CD200-induced Tregs were reportedly sufficient in suppressing T cell proliferation but appeared to have no impact in modulating Th1 cytokine release (TNF-α, IFN-γ, and IL-2) based on Treg depletion experiments [53].

#### 3.1.4. Expansion of MDSCs

CD200/CD200R signaling has also been suggested to mediate immunosuppression by promoting the expansion of myeloid-derived suppressor cells (MDSCs). MDSCs are immature myeloid cells with potent immunosuppressive activity through the release of indoleamine 2,3-dioxygenase, arginase-I, inducible nitric oxide synthase, reactive oxygen species (ROS), and several cytokines, including IL-10, IL-13, and TGF-β [60,61,62,63]. These products function to suppress T cell and NK cell antitumor responses while simultaneously promoting the expansion of other immunosuppressive immune populations, including Tregs, TAMs, and DCs. In one study testing the hypothesis that CD200 drives the expansion and activity of MDSCs in pancreatic ductal adenocarcinoma (PDAC), an anti-CD200 antibody used to block CD200/CD200R signaling was shown to inhibit PDAC tumor growth in murine models and significantly decrease the levels of tumor infiltrating MDSCs [61]. A concomitant increase in tumor infiltrating CD4+ T cells was also observed. Furthermore, using in vitro studies, CD200 administration synergistically enhanced MDSC expansion when PBMCs were co-cultured with IL-6 and GM-CSF—two reagents known to induce expansion of MDSCs in vitro. This finding was accompanied by increased phosphorylation of STAT3 and decreased expression of IRF-8, a known negative regulator of MDSC expansion (Figure 2F). Moreover, a separate glioma study demonstrated that administration of a CD200R antagonist blocked MDSC expansion in glioma tumors and reversed CD200/CD200R-mediated immune suppression. This was accompanied by elevated CD8+ T cell counts and enhanced TNF-α and IFN-γ production [64]. Taken together, these research findings suggest a role for CD200/CD200R signaling in promoting the expansion of MDSCs and subsequent immunosuppression in tumors. However, further analysis of the downstream CD200/CD200R signaling pathways is necessary to elucidate the underlying mechanisms driving MDSC expansion.

#### 3.1.5. CD200/CD200R Signaling Regulates Chemokine Expression Levels in TAMCs

In keeping with its immunosuppressive role, CD200/CD200R signaling promotes an unfavorable TME by differentially regulating chemokine release from TAMCs. These signaling molecules dictate the types of immunosuppressive and/or effector immune cell subsets that are recruited and comprise the TME, thus influencing the anti-tumor immune response. In studies using neuroblastoma and melanoma murine models, the absence of CD200/CD200R signaling (using CD200R- mice) caused TAMCs to upregulate CCL24 and CCL8 levels but downregulate the production of CXCL3, CXCL2, and CCL3 [65]. This process occurs due to tumor-induced activation of ERK and p38 MAP kinases and operates independently of STAT and NF-kB signaling (Figure 2G) [66]. These findings are relevant because CCL24 (eotaxin-2) functions as a chemotactic agent for the recruitment of eosinophils [67]. Although the exact role of eosinophils in cancer is not fully understood, some studies suggest they contribute towards anti-tumor immunity by inducing tumor death via the production of anti-tumor factors such as TNF-α, along with recruiting CD8+ T cells into tumors via CXCL9 and CXCL10 release [68,69]. These CD8+ T cells, in turn, directly facilitate tumor death and produce TNF-α and IFN-γ to further activate eosinophils, thus creating a positive feedback loop to promote their anti-tumor activity [69,70]. In addition, CCL8 (MCP-2) functions to recruit monocytes, T lymphocytes, NK cells, basophils, mast cells, and eosinophils, which contribute towards increased infiltration of immune effector cells [71]. Furthermore, CXCL3, CXCL2, and CCL3 are known to interact with the chemokine receptor, CXCR2, on neutrophils, which aids in the recruitment and tumor-infiltration of additional neutrophils [65,72,73]. These immune cells induce tumor-associated inflammation and angiogenesis, which contributes to tumor progression, diminished T cell responses, and reduced recruitment of other immune effector cells [14,74,75,76,77]. Thus, the production of CCL24 and CCL8, and the reduction of CXCL3, CXCL2, and CCL3 in TAMCs, explain why mice lacking intact CD200/CD200R signaling more potently rejected neuroblastoma and melanoma tumors relative to wild-type conditions [65]. This serves as additional evidence illustrating how CD200/CD200R signaling can exert its pro-tumorigenic effects.

#### 3.1.6. CD200 Facilitates M2 Macrophage Polarization through β-catenin/S100a4-RAGE/NF-𝜅B/M-CSF Signaling

CD200 has also been demonstrated to exert pro-tumor effects through intracellular signaling cascades within tumor cells that operate independently of CD200R. According to Shin et al., CD200 expression in head and neck squamous cell carcinoma (HSNCC) tumors was reported to upregulate the expression of immune-related genes, including macrophage colony-stimulating factor (M-CSF) [23]. M-CSF functions as a cytokine that facilitates the polarization of macrophages towards the M2, tumor-promoting phenotype in the TME, which has been associated with the secretion of anti-inflammatory molecules like IL-10 and TGFβ1, the release of VEGF, and the production of enzymes implicated in the suppression of adaptive immunity such as indoleamine-2,3-dioxygenase (IDO) and arginase- 1 [78,79,80,81]. Mechanistically, CD200′s influence on M-CSF expression levels is believed to operate via activation of the β-catenin/S100a4-RAGE/NF-𝜅B/M-CSF axis (Figure 2H). Specifically, following cleavage of the CD200 cytoplasmic tail by γ-secretase, the free CD200 domain binds with β-catenin [82]. This activates the S100A4/RAGE pathway, which then induces signaling through the NF-𝜅B pathway [83]. This culminates in enhanced transcription of M-CSF, which can induce M2 polarization of macrophages and permit them to exert their pro-tumor effects [84]. Furthermore, Shin et al. reported that CD200 blockade using sCD200R1-Ig-expressing adenovirus (the Fc domain of an antibody fused with the CD200R extracellular domain) not only diminished the activity of the β-catenin/S100a4-RAGE/NF-𝜅B/M-CSF signaling pathway and inhibited growth of CD200-expressing HNSCC tumors, but also reversed the polarization of M2 macrophages back to an M1 phenotype. These M1 macrophages are known to enhance T cell infiltration and activation, which can aid in the anti-tumor immune response. However, they also exert tumoricidal effects and produce pro-inflammatory cytokines, including IL1β, IL-6, TNF-α, IL-12, and IL-23, thus promoting a Th1 cytokine profile and stimulating the cytotoxic activity of T lymphocytes and NK cells [85]. As such, these research findings suggest yet another tumor-promoting mechanism of CD200.

#### 3.1.7. CD200/CD200R Signaling Facilitates cSCC Invasion and Metastasis via Ctsk Expression

Aside from its role in suppressing anti-tumor immunity, the CD200/CD200R axis has also been hypothesized to mediate cancer invasion and metastasis by facilitating the remodeling and degradation of extracellular matrix proteins. Using cutaneous squamous cell carcinoma (cSCC) models, Khan et al. demonstrated that CD200/CD200R signaling upregulates the expression of collagen proteinase Cathepsin K (Ctsk) from tumor infiltrating myeloid lineages in the TME, including CD200R-expressing MDSCs and TAMs (Figure 2I) [86]. Ctsk functions as a collagenolytic peptidase and an osteoclast factor that is involved in bulk collagen degradation and bone resorption. In the skin, Type I collagen bundles comprise the majority of connective tissue components that support the epidermis above. These bundles serve as the primary physical impediment that obstructs cSCC cell invasion through the skin and the subsequent metastasis. Because of Ctsk’s unique ability to recognize and sever multiple cleavage sites in the triple helix of these collagen bundles, Ctsk expression is believed to be required for adequate connective tissue degradation to permit cSCC cell invasion and metastasis [87,88,89,90]. Thus, although the exact mechanism linking CD200/CD200R signaling activity with Ctsk expression remains unknown, engagement of the CD200/CD200R axis is believed to stimulate cSCC cell invasion and metastasis through Ctsk upregulation.

#### 3.1.8. CD200 Induces Epithelial-to-Mesenchymal Transition

CD200 expression has been implicated in cancer progression, particularly through the induction of epithelial-to-mesenchymal transition (EMT). EMT refers to the biological process where a polarized epithelial cell undergoes biochemical changes to acquire a mesenchymal cell phenotype characterized by enhanced migratory abilities, invasiveness, resistance to apoptosis, and production of ECM components [91]. Moreover, EMT has been associated with cancer stemness, metastasis, and recurrence along with resistance to chemoradiotherapy [92,93]. According to Shin et al., CD200 functions as an EMT driver by upregulating the expression of EMT-related genes, including N-cadherin and vimentin, in HNSCC cell lines, while downregulating E-cadherin (epithelial marker). These effects were neutralized by CD200 knockdown and blockade. The molecular mechanism underlying CD200-driven EMT involves a “noncanonical” CD200 signaling pathway that functions independently of CD200R [21]. Specifically, due to an unknown stimulus, the CD200 cytoplasmic tail gets cleaved by γ-secretase and interacts with β-catenin. This CD200-cytoplasmic-domain/β-catenin complex subsequently translocates into the nucleus and increases the expression of EMT-related genes (Figure 1J) [22]. Thus, to inhibit cancer progression, strategies to block this CD200 “non-canonical” pathway using sCD200R1 or anti-CD200 monoclonal antibodies may yield favorable outcomes in the clinical setting.

Furthermore, CD200 expression also promoted EMT in bladder cancer cells. According to Wu et al., CD4+ exhausted T cells with high CD200/PD-1 expression levels exhibited higher expression of EMT transcription factors, including CDH11, DCN, ZEB1, ZEB2, TWIST1, and SNAI1, relative to other CD4+ T cell subclusters. In fact, this ability to induce EMT-related genes can be harvested by malignant bladder cancer cells to facilitate cancer progression, and this occurs through the GAS6-AXL axis—a signaling pathway previously implicated in tumor cell proliferation, EMT, and immune evasion [94]. Because GAS6 has the capacity to stimulate AXL-mediated chemotaxis, malignant bladder cancer cells can utilize GAS6-AXL signaling to recruit PD1hi CD200hi CD4+ exhausted T cells and indirectly promote EMT. Furthermore, bladder cancer cells have the capacity to upregulate GAS6 expression by modifying m6A via METTL3 activity. This prevents m6A from being able to suppress GAS6-mediated effects, thereby promoting the transcription of EMT-related genes and inducing cancer progression [94,95].

### 3.2. CD200/CD200R Mechanisms for Inhibiting Tumor Growth and Metastasis

Although ample evidence exists linking the CD200/CD200R axis to immunosuppression and cancer progression, current studies have also shown an anti-tumorigenic function associated with this pathway. In a 4THM breast carcinoma murine model, CD200 overexpression in CD200 transgenic and CD200R1 knockout BALB/c mice was correlated with the complete regression of primary tumors in 3/7 CD200 transgenic mice and attenuated visceral metastasis to the lungs and liver. On the other hand, inhibiting CD200/CD200R signaling through CD200R depletion was associated with augmented metastasis to the lungs and liver in CD200R KO mice relative to wild type [15]. In a B16 melanoma study, inoculation with CD200+ B16 melanoma cells disrupted tumor formation and growth in C57BL/6 mice and significantly reduced metastatic tumor foci formation in the lungs. Moreover, in CD200R-deficient mice that were implanted with B16-CD200 melanoma cells, enhanced growth of CD200+ tumors was observed along with metastasis to the liver, lungs, kidneys, and peritoneal cavity [16]. Using an E.G7 T cell lymphoma cell line and MC38-OVA epithelium-derived tumor model, co-inoculation with CD200+ CTLs prevented tumor formation in three of four mice and two of four mice, respectively. CD200 was also shown to have a favorable role in non-small cell lung cancer (NSCLC) where higher levels of tumoral CD200 expression were correlated with significantly improved overall survival, recurrence-free survival, and cancer-specific survival relative to those with low tumoral CD200 expression [96]. These findings contradict the existing paradigm and understanding of CD200 as a tumor-promoting molecule and may indicate a lack of universality of CD200 function across human cancers. Thus, contrary to earlier conclusions, CD200 may have a dichotomous role in differentially regulating tumor growth, progression, and metastasis based on cancer type. We now discuss these studies and the proposed anti-tumorigenic mechanisms of CD200 in facilitating cancer growth and metastasis.

#### 3.2.1. CD200 Inhibits Tumor Progression by Restricting the Inflammatory Tumor Microenvironment

The bidirectional role of CD200 may be attributed to the inflammatory microenvironment of the specific tumor. One of the hallmarks of cancer is tumor-promoting inflammation—a non-resolving and chronic process that occurs due to tumor-induced necrosis of healthy cells [11]. This results in chronic activation of the immune system, which can subsequently trigger carcinogenesis through the creation of an inflammatory microenvironment, increasing DNA damage, genomic instability, and malignant transformation. In many cases, the degree of inflammation causes a proportionate increase in tumorigenesis and the aggressiveness of carcinomas. For example, melanoma and NSCLC are considered “hot tumors,” characterized by a T-cell-inflamed tumor phenotype enriched with infiltrating T lymphocytes, elevated IFN-γ signaling, and increased PD-L1 expression. These tumors are also genomically unstable with a high tumor mutation burden (TMB) and an elevated prevalence of somatic mutations in their genomes, creating more neoantigens that the immune system can recognize and mount an antitumor response against [10]. Tumors with these inherent properties have a high probability of responding favorably to immune checkpoint inhibitors and may also be responsible for the anti-tumorigenic function associated with CD200.

**Figure 3:**
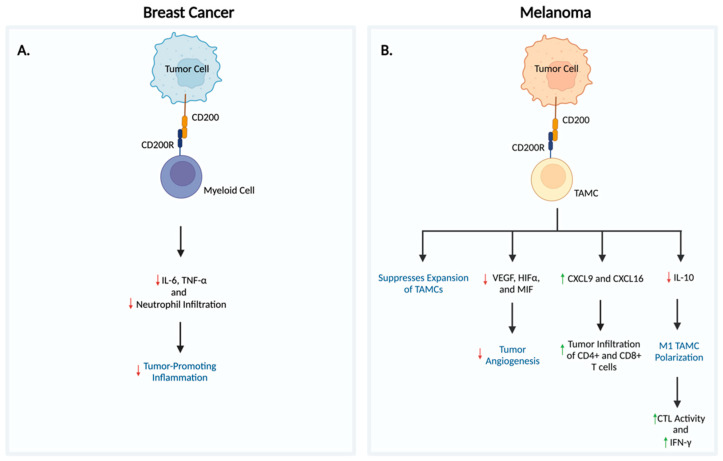
**Proposed mechanisms elucidating the anti-tumorigenic role of CD200/CD200R signaling in breast carcinoma and melanoma**. The protective, anti-tumorigenic role of CD200/CD200R signaling has primarily been reported in breast carcinoma and melanoma studies. (**A**) In breast carcinoma, the CD200/CD200R axis prevents tumor-promoting inflammation via decreased tumor infiltration of neutrophils and reduced production of IL-6 and TNF-α. In contrast, (**B**) CD200/CD200R signaling in melanoma suppresses the expansion of TAMCs, inhibits VEGF, HIFα, and MIF-mediated tumor angiogenesis, facilitates tumor infiltration of CD4+ and CD8+ T cells through increased CXCL9 and CXCL16 production, and polarizes macrophages to the M1 phenotype by decreasing IL-10 release. “https://www.biorender.com/ (accessed on 1 October 2023)”.

In this respect, while CD200 is generally considered to be immunosuppressive, its anti-tumorigenic function may be explained by its potent anti-inflammatory effects (Figure 3A). For instance, using the highly aggressive and inflammatory 4THM breast carcinoma murine model, Erin et al. demonstrated that CD200 overexpression limited neutrophil infiltration of tissues and decreased production of the inflammatory cytokines: IL-6 and TNF-α. Exogenous exposure to CD200fc also suppressed IL-6 production. IL-6 is commonly expressed by cancer stem cells (CSCs) and is strongly correlated with both tumor stage and poor prognosis. Similarly, TNF-α possesses tumor-promoting capabilities by inducing hemorrhagic necrosis [12]. These inflammatory mediators produced by CSCs have the capacity to modify the tumor microenvironment and induce inflammation, perpetuating the growth of CSCs [97,98]. Thus, based on these findings, CD200 expression may exert its anti-metastatic/antitumoral effects, particularly in highly aggressive and inflammatory carcinomas, by inhibiting IL-6 and TNF-α-mediated inflammation while also limiting tissue infiltration by neutrophils.

#### 3.2.2. CD200 Limits the Expansion of Tumor-Associated Myeloid Cells

The CD200/CD200R interaction has also been suggested to exert antitumoral and antimetastatic effects by altering the populations of TAMCs and inhibiting their functions, thus shaping the TME and influencing T cell responses in tumors. TAMCs, including MDSCs and TAMs, express high levels of CD200R and have been posited to play significant roles in inducing tumor initiation, formation, progression, and metastasis by releasing pro-angiogenic factors, aiding in extracellular matrix breakdown, and suppressing anti-tumor immunity. These myeloid cells are believed to interact directly with tumor cells via the CD200/CD200R interaction, making them susceptible to CD200-mediated inhibition (Figure 2B) [13,99]. As demonstrated in melanoma studies, TAMC levels were inversely proportional to CD200 expression, suggesting that CD200/CD200R signaling suppresses the expansion of TAMCs. However, when CD200/CD200R signaling was disrupted, CD11b^+^Gr1^+^, CD11b^+^Ly6C^+^, and CD11b^+^Ly6C^−^ TAMC populations expanded along with increased expression of VEGF, HIF1α, and migration inhibitory factor (MIF) genes. These products facilitate tumor angiogenesis along with tumor growth/progression. Furthermore, CD200/CD200R signaling may also differentially regulate the expression of CXCL9, CXCL16, and CCL8 [99,100,101,102]. CXCL9 and CXCL16 are chemokines involved in recruiting T cells and facilitating tumor infiltration. When CD200/CD200R signaling was disrupted, CXCL9 and CXCL16 expression levels not only decreased but there were significantly reduced numbers of CD4+ and CD8+ T cells in the TME, resulting in an impaired immune response. On the other hand, CCL8 production was upregulated in the absence of CD200/CD200R signaling [103]. Although CCL8 has been known to recruit immune cells during the immune response, studies have reported that tumoral CCL8 expression by TAMCs promotes a TME that favors metastasis of cervical cancer, facilitates the migration/invasion of esophageal squamous cell carcinoma, recruits Tregs to the TME, and augments stem-like features in glioblastoma. These secondary CCL8 effects are additional mechanisms believed to drive tumor growth in the absence of CD200/CD200R signaling [71,103,104,105,106].

In addition, a separate melanoma study concluded that tumoral expression of CD200 similarly alters the tumor microenvironment by inhibiting IL-10 production from TAMCs [107]. IL-10 is a cytokine commonly produced by TAMs and MDSCs and serves to suppress CTL effector functions. Moreover, Il-10 has been shown to induce the polarization from M1 macrophages to the M2 phenotype (Figure 3B) while simultaneously promoting tumor growth, invasion, and angiogenesis [108,109]. In this respect, by inhibiting IL-10 production, CD200 tumoral expression may serve a favorable role in melanoma by facilitating the transition of TAMCs to the M1 phenotype. These M1 “re-educated” TAMCs have been shown to potently kill cancer cells [107]. In addition, when coupled with adoptively transferred CTL therapy, this study reported that CD200 expression in melanoma cells permitted better infiltration of activated and tumor specific CTLs. These have the capacity to target antigenic tumor and stromal cells through IFN-γ production, leading to tumor rejection and reduced tumor recurrence [102,110,111,112].

## 4. Current and Potential Clinical Applications of CD200/CD200R in Immunotherapy

Despite the uncertainty over a definitive role of CD200/CD200R in tumorigenesis, the CD200/CD200R axis has garnered interest as a key factor in modulating the efficacy of immunotherapy and targeted therapy and has emerged as a promising target for blockade (Table 1).

### 4.1. CD200/CD200R Blockade

Between June 2008 and December 2010, a Phase I clinical trial (NCT00648739) was conducted to evaluate the safety, pharmacology, and therapeutic efficacy of CD200/CD200R blockade in advanced CLL and multiple myeloma (MM) patients. Using Samalizumab, a recombinant humanized anti-CD200 monoclonal antibody, this study found that CD200/CD200R blockade restored CTL-mediated anti-tumor activity and reduced tumor burden in 14/23 CLL patients, though 16 patients achieved stable disease. However, all three MM patients in this study showed disease progression, and a range of adverse reactions related to Samalizumab treatment were documented, including anemia, neutropenia, thrombocytopenia, reduced visual acuity, muscular weakness, allergic reactions, and urticaria. Despite this, Samalizumab was observed to cause a dose-dependent decline in both CD200 expression and levels of circulating CD200+ CD4+ T cells, with higher doses (300–600 mg/m^2^) being associated with sustained responses. Samalizumab was also deemed to have a favorable safety profile, with doses between 50–600 mg/m^2^ being well tolerated by patients [56]. This preliminary data provides insights into the potential therapeutic benefit of CD200-targeted inhibition in CLL patients and warrants further clinical experimentation using other hematologic cancer types and higher dosing regimens.

CD200/CD200R blockade has been further investigated in in vivo murine models using the humanized anti-CD200 antibody TTI-CD200. TTI-CD200 treatment has been shown to induce the transition towards a Th1 cytokine profile characterized by increased IL-2 and IFN-γ production in AML and ALL studies. According to Diamanti et al., TTI-CD200 administration induced a 19-fold increase in IL-2 production from ALL cells with high CD200 expression, indicating a release of CD200-mediated IL-2 suppression. Furthermore, Rastogi et al. reported increased IFN-γ secretion from NK cells after treating high CD200-expressing AML cells with TTI-CD200. These alterations in chemokine levels may explain why TTI-CD200 treatment mediated a delay in disease progression, a significant decrease in disease burden, and an extension in survival for mice inoculated with CD200-expressing ALL cells from “low-risk” patients [113].

### 4.2. Synergistic Effects of CD200 and/or CD200R on Other Cancer Therapies

Aside from direct CD200/CD200R blockade using monoclonal antibodies, CD200/CD200R signaling has also been investigated in combination with other cancer therapies. Interestingly, these studies have demonstrated a synergistic role of CD200 and/or CD200R in amplifying the anti-tumor effects of these therapies.

For instance, the administration of an anti-CD200R agonist (OX110) potentiated the anti-tumor effects of R848, a Toll-like receptor 7 (TLR7) agonist, and significantly reduced tumor volume and growth in colon carcinoma murine models. This finding occurred due to a shift in the phenotype and composition of intratumoral myeloid cells. Specifically, dual administration of R848 and OX110 caused the TME to transition from a dominant MHC-II^+^ immature macrophage population to a monocytic/MHC-II^−^ immature macrophage population. This was accompanied by reduced expression of macrophage markers, including CD206 (M2/TAM marker), CD86 (macrophage activation marker), CD115 (M-CSF receptor), and F4/80, along with significantly decreased production of IL1β from tumor-derived myeloid cells. Collectively, these results indicate an inhibition of macrophage maturation (including M2-like TAMs), which impairs these myeloid cells’ tumor-promoting properties. Signaling through CD200R likely mediates this process. Furthermore, Pilch et al. speculated that TLR7 stimulation can subsequently induce cell-mediated immunity and exert its proinflammatory effects through the production of type 1 IFNs, the polarization of tumor-infiltrating macrophages towards the M1 phenotype, and the release of the inflammatory cytokine IL-6 [114,115,116]. In this manner, these TLR7-mediated pro-inflammatory effects would synergize with the anti-CD200R agonist to facilitate anti-tumor responses.

Moreover, Ishibashi et al. demonstrated that CD200 expression on cancer-associated fibroblasts (CAFs) increased the efficacy of gefitinib treatment, an epidermal growth factor receptor tyrosine kinase inhibitor (EGFR-TKI) and amplified its apoptotic effects on lung adenocarcinoma cells bearing EGFR mutations. This was evidenced by decreased cell viability of lung adenocarcinoma cells when CD200+ CAFs were cocultured with gefitinib. However, this antitumor effect was diminished upon CD200 knockdown. In fact, these results paralleled clinical observations, as lung cancer patients with CD200+ CAFs experienced longer progression-free survival following gefitinib treatment relative to patients with CD200− CAFs. With these results in mind, therapeutic strategies to upregulate CD200 signaling may be effective in treating EGFR-mutant NSCLC tumors by re-sensitizing them to gefitinib and potentially overcoming EGFR-TKI resistance. Of note, Ishibashi et al. hypothesized that CD200 may exert these antitumor effects independently of CD200R since this receptor was not expressed in this study’s cell line. Thus, CD200 signaling through an unknown receptor is responsible for triggering the observed pro-apoptotic signaling cascade upon EGFR-TKI treatment [117].

CD200 expression on cytotoxic T lymphocytes (CTLs) has also been associated with the efficacy of anti-PD-1/PD-L1 therapy. Using a cohort of patients with basal cell carcinoma and squamous cell carcinoma, Wang et al. demonstrated that anti-PD-1 therapy triggered the expansion of intra-tumoral CD200+ T cells, and higher proportions of these CD200+ TIL subsets were positively associated with favorable clinical outcomes to ICB (immune checkpoint blockade) therapy. This pattern was consistent with studies using colon cancer murine models. Furthermore, to determine whether CD200+ CTLs were necessary for efficacious anti-PD-L1 treatment, Wang et al. depleted CD200+ CTLs using anti-CD200 antibodies in vivo. This reduced the efficacy of anti-PD-L1 treatment in inhibiting tumor growth, indicating that CD200 expression is necessary for effective ICB therapy. In fact, these findings can be attributed to the polyfunctionality of CD200+ CTLs. Relative to CD200− CTLs, CD200+ CTLs exhibit superior proliferative abilities, higher expression of activation markers, and greater production of effector molecules, including CD107a, IFN-γ, TNF-α, and granzyme B (GzmB). These functions enable CD200+ CTLs to exert their neoantigen-specific cytolytic activities to a higher degree and ultimately induce tumor cell death. Moreover, an analysis of the epigenetics of CD200+ CTLs can explain this polyfunctionality. According to Wang et al., CD200+ CTLs exhibited increased chromatin accessibility and gene activity for genes encoding IFN-γ, TNF-α, and GzmB relative to CD200− CTLs. This was accompanied by elevated signaling through RUNX3 and AP1, indicating that these pathways may be responsible for maintaining CD200+ CTL effector functions. Finally, using the RNA-seq/survival data from six publicly available patient cohorts who received ICB therapy, Wang et al. demonstrated that high CD200+ CTL tumor infiltration was indicative of a favorable response to ICB therapy, regardless of tumor type. Collectively, these findings support the notion that CD200+ CTLs in the TME are essential for efficacious anti-PD-1/PD-L1 therapy [17].

Another study employed double knock-in humanized mice to explore a novel immunotherapy approach using a combination of PD-1 and CD200R-based therapies in tumor models. While they confirmed the efficacy of nivolumab in vitro and observed trends toward more complete responses and changes in immune cell populations in some tumor models, overall survival did not improve significantly. The research highlighted potential limitations, including variable CD200 expression on tumor cells and differences in tumor microenvironments between subcutaneous and other tumor models. The role of CD200R in cancer remains uncertain due to conflicting findings in mouse and human studies, with some supporting a pro-tumorigenic role. The study suggests further investigation into this combination therapy, particularly in models with higher CD200 expression [118].

## 5. Future Directions

As we move forward in exploring the role of the CD200/CD200R axis in cancer, several future directions emerge:

Mechanistic insights: Identifying reliable biomarkers, such as CD200 expression levels, that predict responses to CD200-targeted therapies will be crucial. This will help personalize treatment strategies and select patients who are most likely to benefit. Continued research into the underlying mechanisms through which CD200 influences immune responses and tumor progression is essential. Understanding the signaling pathways and interactions involved can lead to the development of more targeted therapies. Studying the impact of CD200 on the tumor microenvironment and its dynamic changes throughout tumor progression will also provide insights into the context-specific roles of CD200 in cancer.

Clinical trials: Further clinical trials are needed to evaluate the safety and efficacy of CD200/CD200R blockade in various cancer types. These trials should investigate optimal dosing regimens, patient selection criteria, and potential combination therapies to maximize therapeutic benefits.

Combination therapies: Investigating the synergistic effects of combining CD200-targeted therapies with other immunotherapies, such as checkpoint inhibitors or targeted therapies, holds promise for enhancing treatment outcomes.

Personalized medicine: Integrating CD200-related information into precision medicine approaches can aid in tailoring treatment plans for individual cancer patients, optimizing therapeutic responses and minimizing adverse effects.

Exploration of other immunotherapies: While PD-1/PD-L1 and CTLA-4 have been extensively studied, investigating the interplay between CD200 and other immune checkpoints such as LAG-3, TIM-3, and VISTA, and cytokines such as TNFs may uncover additional therapeutic opportunities [1,9,119,120].

In summary, the CD200/CD200R axis is a multifaceted player in the immune landscape of cancer, with the potential to both promote and inhibit tumor growth. The ongoing exploration of CD200’s roles and therapeutic implications offers hope for improved cancer treatments and underscores the complexity of immune regulation in the fight against cancer.

## 6. Conclusions

The CD200/CD200R axis plays a complex and bidirectional role in cancer, regulating immune responses and impacting tumor progression. While CD200 is traditionally associated with immunosuppression and poor clinical outcomes, recent studies have revealed its diverse effects on different cancer types, stages, and microenvironments. CD200’s ability to modulate inflammatory responses, influence the expansion of tumor-associated myeloid cells, and regulate T cell functions underscores its significance in the context of cancer biology. The intricate interplay between CD200 and its receptor, CD200R, has prompted extensive research into potential therapeutic strategies for cancer treatment. Blockade of CD200/CD200R interactions using monoclonal antibodies like Samalizumab and TTI-CD200 has shown promise in restoring anti-tumor immunity, particularly in hematologic malignancies. Additionally, CD200 expression in CAFs has demonstrated enhanced sensitivity to certain targeted therapies, such as EGFR-TKIs, offering new avenues for combination treatments. Furthermore, anti-CD200R agonists have proven effective in reshaping the tumor microenvironment and improving the anti-tumor effects of other immunotherapies, such as TLR7 agonists. The interaction between CD200 and immune checkpoint molecules like PD-1/PD-L1 has also garnered attention, with evidence suggesting that CD200 expression can influence the efficacy of PD-1/PD-L1 blockade. Understanding the intricate network of checkpoint molecules and their impact on immunotherapy response remains a critical area of research.

## Figures and Tables

**Table 1:** The clinical applications of CD200/CD200R in immunotherapy.

Treatment	Mechanism/Effects	Progress
Samalizumab	Restore CTL-mediated anti-tumor activity.	Phase I Clinical Trial
TTI-CD200	Increase IL-2 and IFN-γ production.	Pre-Clinical Study
OX110+ R848	Shift TME from MHC-II+ immature macrophage population to MHC-II- immature macrophage population and induce cell-mediated immunity.	Pre-Clinical Study
CD200+ Gefitnib	Reduce the viability of CAF and amplify the apoptotic effects of lung adenocarcinoma cells bearing EGFR mutations.	Pre-Clinical Study
CD200+ Anti-PD-1/PD-L	CD200+ CTL increases the efficacy of anti-PD-1/PD-L1 therapy and increases chromatin accessibility and activity of IFN-γ, TNF-α, and GzmB genes.	Pre-Clinical Study
CD200+ Nivolumab	Improves immune response but not necessarily overall survival.	Pre-Clinical Study
